# High Resolution Typing by Whole Genome Mapping Enables Discrimination of LA-MRSA (CC398) Strains and Identification of Transmission Events

**DOI:** 10.1371/journal.pone.0066493

**Published:** 2013-06-21

**Authors:** Thijs Bosch, Erwin Verkade, Martijn van Luit, Bruno Pot, Paul Vauterin, Ronald Burggrave, Paul Savelkoul, Jan Kluytmans, Leo Schouls

**Affiliations:** 1 Laboratory for Infectious Diseases and Screening, National Institute for Public Health and the Environment (RIVM), Bilthoven, The Netherlands; 2 Laboratory for Microbiology and Infection Control, Amphia Hospital, Breda, The Netherlands; 3 Laboratory for Medical Microbiology and Immunology, St. Elisabeth Hospital, Tilburg, The Netherlands; 4 Applied Maths, Sint-Martens-Latem, Belgium; 5 Piext B.V., Rosmalen, The Netherlands; 6 Department of Medical Microbiology, Academic Hospital Maastricht, Maastricht, The Netherlands; 7 Department of Medical Microbiology, VU University Medical Center, Amsterdam, The Netherlands; University Hospital Münster, Germany

## Abstract

After its emergence in 2003, a livestock-associated (LA-)MRSA clade (CC398) has caused an impressive increase in the number of isolates submitted for the Dutch national MRSA surveillance and now comprises 40% of all isolates. The currently used molecular typing techniques have limited discriminatory power for this MRSA clade, which hampers studies on the origin and transmission routes. Recently, a new molecular analysis technique named whole genome mapping was introduced. This method creates high-resolution, ordered whole genome restriction maps that may have potential for strain typing. In this study, we assessed and validated the capability of whole genome mapping to differentiate LA-MRSA isolates. Multiple validation experiments showed that whole genome mapping produced highly reproducible results. Assessment of the technique on two well-documented MRSA outbreaks showed that whole genome mapping was able to confirm one outbreak, but revealed major differences between the maps of a second, indicating that not all isolates belonged to this outbreak. Whole genome mapping of LA-MRSA isolates that were epidemiologically unlinked provided a much higher discriminatory power than *spa*-typing or MLVA. In contrast, maps created from LA-MRSA isolates obtained during a proven LA-MRSA outbreak were nearly indistinguishable showing that transmission of LA-MRSA can be detected by whole genome mapping. Finally, whole genome maps of LA-MRSA isolates originating from two unrelated veterinarians and their household members showed that veterinarians may carry and transmit different LA-MRSA strains at the same time. No such conclusions could be drawn based *spa*-typing and MLVA. Although PFGE seems to be suitable for molecular typing of LA-MRSA, WGM provides a much higher discriminatory power. Furthermore, whole genome mapping can provide a comparison with other maps within 2 days after the bacterial culture is received, making it suitable to investigate transmission events and outbreaks caused by LA-MRSA.

## Introduction

*Staphylococcus aureus* and in particular methicillin resistant *S. aureus* (MRSA) is a bacterial pathogen that is associated with serious hospital- and community-acquired infections [1], [2]. In the Netherlands, the incidence of MRSA infections is still low due to the restricted use of antibiotics and the successful implementation of the ‘search and destroy’ policy. However, the number of MRSA isolates sent to the national institute for public health and the environment (RIVM) in the context of the national MRSA surveillance, has been gradually increasing in the last years [3]. This increase is mainly caused by the emergence of a new single MRSA clade multi-locus sequence type ST398 originating from livestock, mainly pigs. ST398 was first described by Voss et al. in 2005 and since then ST398 has been found in numerous countries worldwide [4]–[8]. ST398 has been isolated from different types of domesticized animals and therefore is ST398 also known as livestock-associated MRSA (LA-MRSA) [9], [10]. After the first reports in 2005, LA-MRSA (ST398) has spread very rapidly in the Netherlands and has become the predominant MRSA clade since 2007. In 2010, 38% of all isolates sent to the RIVM were LA-MRSA [3].

Typing of LA-MRSA however, has turned out to be a challenge. One of its characteristics is that LA-MRSA are non-typeable with regular pulsed-field gel electrophoresis (PFGE) due to methylation of the *Sma*I recognition site [11]. In recent years, a number of reports have shown that PFGE with restriction enzyme *Cfr*9I, a neoschizomer of *Sma*I, can be used to overcome the problems with DNA-methylation [12], [13]. Although PFGE with *Cfr*9I yields a relatively high discriminatory power for LA-MRSA isolates, PFGE remains a time-consuming, laborious and non-portable method. Other typing techniques, such as staphylococcal protein A (*spa*)-typing and multiple-locus variable number of tandem repeat analysis (MLVA), can be used to characterize LA-MRSA, but yield very limited discrimination within this clade. From 2008–2012, 17,869 MRSA isolates were characterized by *spa*-typing and MLVA within our national MRSA surveillance. The predominant MLVA complex was MC398, representing LA-MRSA and comprising of 7,066 isolates. Although 96 different *spa*-types and 78 different MLVA-types (MT) were found within this clade, the 2 dominant types, *spa*-type t011, MT398 (*n* = 4093) and *spa*-type t108, MT572 (*n* = 1282), accounted for 76% of all LA-MRSA isolates. In contrast, MC5 (MRSA, *n* = 2520), the most isolated clade after MC398, yielded 113 different *spa*-types and 205 different MLVA-types with *spa*-type t003, MT130 (*n* = 182) and *spa*-type t002, MT5 (*n* = 126) as the predominant types, but accounting only for 12% of all MC5 isolates. Strains within MC398 show limited variability and the absence of a highly discriminating typing method to characterize MC398 (LA-MRSA) isolates has hampered studies on the origin and transmission routes of this MRSA clade.

In 2007, a molecular analysis technique was introduced initially called optical mapping and now designated as whole genome mapping (WGM), although whole chromosome mapping would be more appropriate. A whole genome map is a high-resolution, ordered, whole genome restriction map and for *S. aureus* isolates these maps consists of 200–300 restriction fragments. In contrast, in PFGE of *S. aureus* only 10–15 non-ordered restriction fragments are used for the analysis [14], [15]. Although Shukla et al. have previously successfully used WGM to characterize MRSA belonging to the USA300 clade [16], the number of reports in which WGM was used for molecular typing of bacterial pathogens is very limited [16]–[18].

In this study, we assessed and validated the capability of whole genome mapping to differentiate LA-MRSA isolates. For this purpose, we used epidemiologically related and non-related MRSA and LA-MRSA isolates.

## Methods

### Strain Selection

For this study a total of 18 MRSA and 45 LA-MRSA isolates were selected to create 84 different whole genome maps (WGMs). Two MRSA strains (NCTC8325, N315) and one LA-MRSA strain (S0385), which are often used as reference strains and for which published genomes are available [25]–[27], were used for reproducibility experiments and comparison of whole genome maps created in our laboratory with *in silico* maps. In addition, two LA-MRSA (MC398) isolated from Dutch veterinarians (VET78 (t = 0 m) and VET35 (t = 0 m)) were used for reproducibility experiments. The capability to identify transmission events was studied using isolates obtained during three well-documented outbreaks in the Netherlands of CA-MRSA (USA300), HA-MRSA (MC45) and LA-MRSA (MC398) and 22 LA-MRSA isolates from Dutch veterinarians and their household members [6], [19], [20]. Finally, 16 LA-MRSA isolates originating from a longitudinal survey of veterinarians frequently visiting livestock farms were used to investigate the discriminatory power of the whole genome mapping method for LA-MRSA (Table 1). All isolates used in this study originated from pre-existing collections and the isolates used to create WGMs were also characterized by MLVA, *spa*-typing and PFGE as described previously [21]–[24].

**Table 1:** Bacterial strains used in this study.

Experiment	MRSA strains	LA-MRSA strains	Reference*
Re-assembly of raw data	NCTC8385	S0385	[25], [27]
Optimal comparison settings	NCTC8385	VET78 (t = 0 m)	[25], VET-study
Stability of WGM	NCTC8385, N315	VET78 (t = 0 m), VET35 (t = 0 m)	[25], [26], VET-study
Comparison with *in-silico* maps	NCTC8385, N315	S0385	[25], [26], [27]
MRSA transmission events	CA-MRSA (*n* = 8), HA-MRSA (*n* = 8)		[19], [20]
Discriminatory power for LA-MRSA		VET-isolates (*n* = 16)	VET-study
LA-MRSA transmission events		Transmission isolates (*n* = 4)	[6]
Suspected LA-MRSA transmission		VET-isolates (*n* = 22)	VET-study

* VET-study, isolates collected for a longitudinal MRSA carriage study among veterinarians and written consent was provided by all participants (E. Verkade personal communication).

### Isolation of HMW DNA

Whole genome mapping requires the input of high molecular weight DNA (HMW DNA) with an average molecule size of approximately 250,000 bp. The Argus™ HMW DNA isolation kit (OpGen, Gaithersburg, USA) provides reagents and a protocol specifically designed for the isolation of HMW DNA. Briefly, a single colony was picked from a plate and suspended in cell wash buffer. Bacteria were treated to form spheroplasts and subsequently lysed to release the HMW DNA. For the isolation of HMW DNA of *S. aureus* this protocol required small but essential adaptations. First, we doubled the amount of lysostaphine (15 units/sample) used during the spheroplasting step and tripled the incubation time (3 hrs) recommended by the manufacturer. Furthermore, to obtain sufficient yield of HMW DNA for WGM we empirically determined that the isolated DNA required to relax and go into solution for at least 24 hrs at room temperature before proceeding to the dilution step. In our protocol, a 1∶40 dilution was usually optimal for *S. aureus*. The quality (e.g. the average molecule size (AMS)) and the concentration of the DNA samples were checked using so-called Quality Control cards (Argus™ QCard kit, OpGen, Gaithersburg, USA). We found that for *S. aureus* a minimum of 5–10 DNA molecules of approximately 250,000 bp should be present per image in order to obtain good WGMs.

### Creating Whole Genome Maps

Whole genome maps were created using the manufacturer’s instructions. Shortly, HMW DNA was applied to Mapcards containing micro channels in which DNA molecules were stretched, bound to a glass surface, and subsequently digested with *Afl*II and stained with a fluorescent agent in a micro fluids system. The restriction fragments were sized in the whole genome mapper and assembled into a whole genome map in which the restriction sites are mapped in the order in which they occur in the chromosome using MapManager software version 1.1 (OpGen). For assembly, only DNA molecules larger than 150,000 bp and with a minimum of 12 restriction sites were included. In a complete map, each assigned restriction site should have been found in at least 30 single molecules (coverage) and typically an average depth of 50 to 80 molecules is found. To assess whether assembly using the settings recommended by OpGen resulted in reproducible maps, the same raw data obtained from DNA of reference strains NCTC8325 and S0385 were assembled into WGMs 5 times each. Comparison of the generated maps revealed identical WGMs demonstrating the reproducibility of the assembly under the recommended conditions. After assembly, the generated restriction maps were imported into MapSolver software version 3.0 (OpGen, Gaithersburg, USA) to create the final circular whole genome map and without further manipulation the map was subsequently saved in .xml file format.

### Analyzing Whole Genome Maps

The .xml files containing the ordered maps were imported into a database created with an alpha version of BioNumerics version 7.0 (Applied Maths, Sint-Martens-Latem, Belgium). For clustering and alignment of the maps, we chose the first restriction fragment after the origin of replication in the chromosome as the starting point of the map, using a map rotation plugin in BioNumerics. For rotation of LA-MRSA maps, the *in silico* whole genome map of LA-MRSA strain S0385 (AM990992) was used as a template and for regular MRSA strains an *in silico* map of NCTC8325 (CP000253) was utilized. The BioNumerics software allowed alignment and clustering of WGMs using filtering of small fragments and size tolerance settings. The background on the algorithms used for the comparisons of whole genome maps in BioNumerics will be described in detail elsewhere. In the alignment, two fragments were considered to be identical if their sizes differed no more than the value set for the absolute tolerance. If this criterion was not met, two fragments were still considered identical if they met the relative tolerance criterion. This relative tolerance is defined as the difference between two fragments dived by their average; (size fragment 1– size fragment 2)/((size fragment 1+ size fragment 2)/2). The similarity between the whole genome maps was calculated by dividing the number of matched fragments by the total number of fragments. The method we chose for cluster analysis was UPGMA. In the BioNumerics software WGMs are represented as linear maps displaying the fragments in randomly chosen colours, with matching fragments sharing the same colour. Fragments of maps that were excluded from the comparison as a result from the fragment filtering were not deleted from the maps, but displayed as blocks with reduced height in the maps.

## Results

### Assessing Optimal Settings for Comparisons

The whole genome mapping encompasses procedures in which the restriction fragments are sized and subsequently assembled into ordered restriction maps. However, there is experimental variation in the sizing and also smaller restriction fragments may variably be lost during the procedure. The reason for this loss is that smaller DNA fragments have a relatively low net charge and as a result a weaker bond to the glass surface of the Mapcard. To compensate for both the variation in sizing and the presence or absence of smaller fragments during clustering and alignment, tolerance and filtering were employed in the BioNumerics software. In order to assess the optimal filtering and tolerance settings, DNA isolated from a MRSA and a LA-MRSA isolate was repeatedly used to create WGMs on 4 consecutive days and analyzed in BioNumerics using an range of filtering and tolerance settings. A combination of a filtering setting that excluded fragments <3,000 bp from the comparison and a relative tolerance of 15% combined with an absolute tolerance of 1,000 bp resulted in maps with a similarity of >99% for replicates of the DNA samples, whilst unrelated samples yielded distinct maps. Settings that were more stringent resulted in artificial differences between maps of the replicates while less stringent settings resulted in loss of the ability to discriminate unrelated samples.

### Stability of Whole Genome Maps and Comparison with in silico Maps

To assess the temporal stability of MRSA genomes as reflected in whole genome maps under laboratory conditions, single colonies from 2 LA-MRSA and 2 MRSA isolates were sub-cultured for 30 consecutive days. The similarity between the WGMs obtained from the DNA isolated on day 1 and day 30 ranged between 99.4% and 100%, showing that under laboratory conditions the MRSA genome was stable enough to yield virtually identical maps.

**Figure 1:**
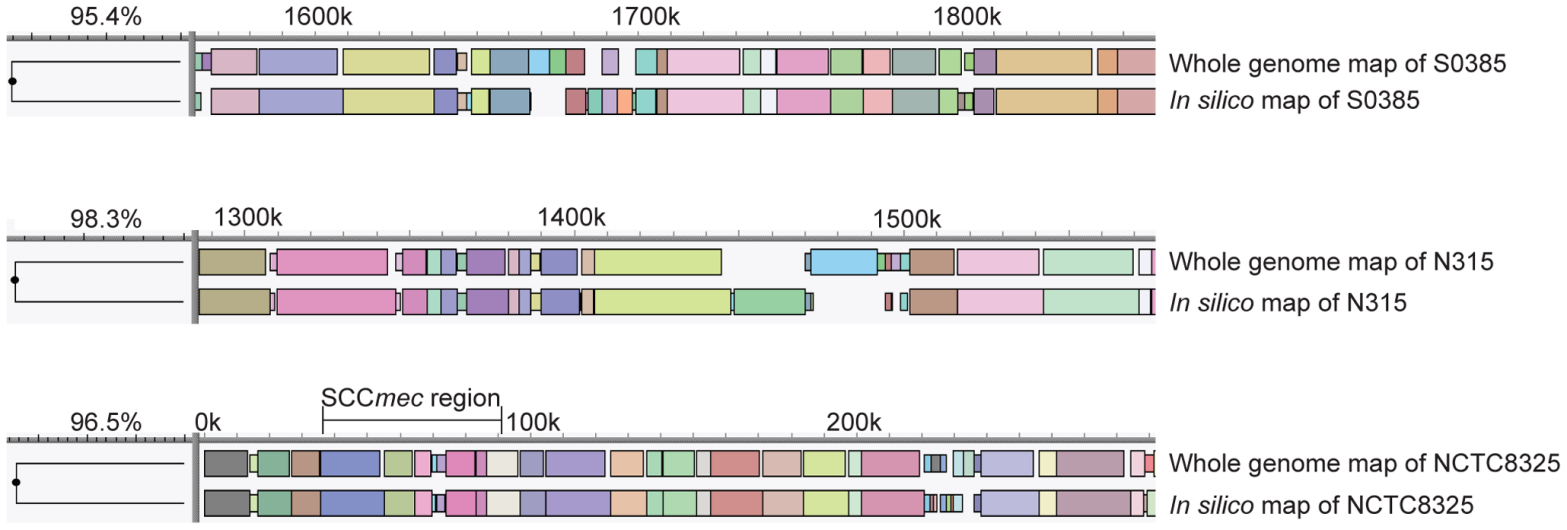
Examples of differences between the WGMs created in the lab and their *in-silico* counterparts. The figure shows details of the comparisons of the maps of LA-MRSA strain S0385 and MRSA strains N315 and NCTC8325. The WGMs are represented as linear maps displaying the fragments in randomly chosen colours, with matching fragments sharing the same colour.

To determine to what extend WGMs accurately reflect the composition of a whole genome sequence, maps obtained from NCTC8325 (MRSA) [25], N315 (MRSA) [26] and S0385 (LA-MRSA) [27] were compared to their *in silico* counterparts generated in the Mapsolver software. The similarity between the WGMs created in the laboratory and their *in silico* counterparts varied between 95.5% and 98.7% for these isolates. Close inspection of the differences between the real WGMs and the *in silico* maps revealed that in general, sizing of the restriction fragments by the whole genome mapper in general results in slightly smaller fragments than those predicted based on the whole genome sequences. Furthermore, there were several locations where the composition of the predicted maps clearly differed from the maps generated in the laboratory (Figure 1).

### Capability of WGM to Identify Transmission Events of MRSA

**Figure 2:**
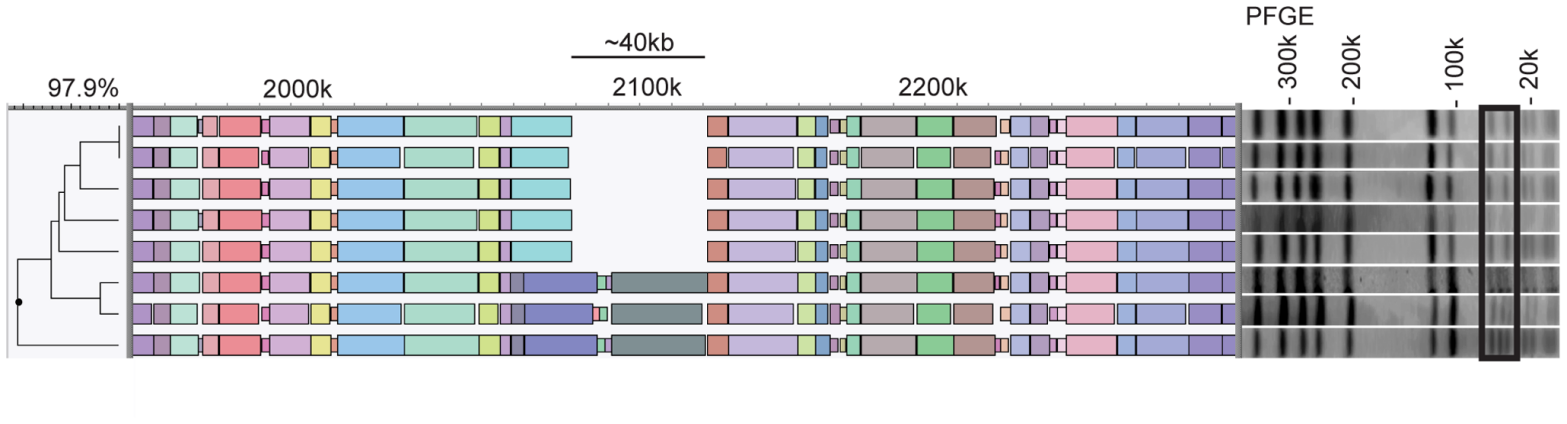
Detail of the whole genome maps of an outbreak of CA-MRSA (USA300) showing an additional DNA segment in 3 isolates. All isolates had *spa*-type t024 and MLVA-type MT308. The gel image on the right hand side shows the PFGE profiles with an additional 80 kb band in the lower 3 isolates.

To determine whether whole genome mapping is capable of identifying transmission events, two earlier reported MRSA outbreaks were investigated. The first outbreak comprised 8 community-acquired MRSA (CA-MRSA) isolates obtained during an outbreak in a Dutch beauty salon in 2006 [19]. According to the authors of this study, PFGE showed that, all isolates belonged to the so-called USA300 cluster and had indistinguishable PFGE banding patterns. However, renewed inspection of the PFGE profiles during our study revealed that 5 isolates had identical PFGE profiles, but 3 isolates had an additional band of approximately 80 kb. Molecular typing characterized all isolates as Panton–Valentine Leukocidin (PVL)-positive, *spa*-type t024 and MT308 (MC8). The whole genome maps of the isolates were also highly similar with a similarity of 97.9% between the most distinct maps. Remarkably, the 3 isolates that had an additional band in PFGE also carried an additional, approximately 40 kb DNA segment which was absent in the 5 other isolates (Figure 2).

Based on the results with the replicates, that yielded >99% similar profiles, and the result of the above described outbreak we chose to set the cut-off value at 98% for indistinguishable profiles. This will allow for the variation in WGMs due to the presence or absence of mobile elements.

**Figure 3:**
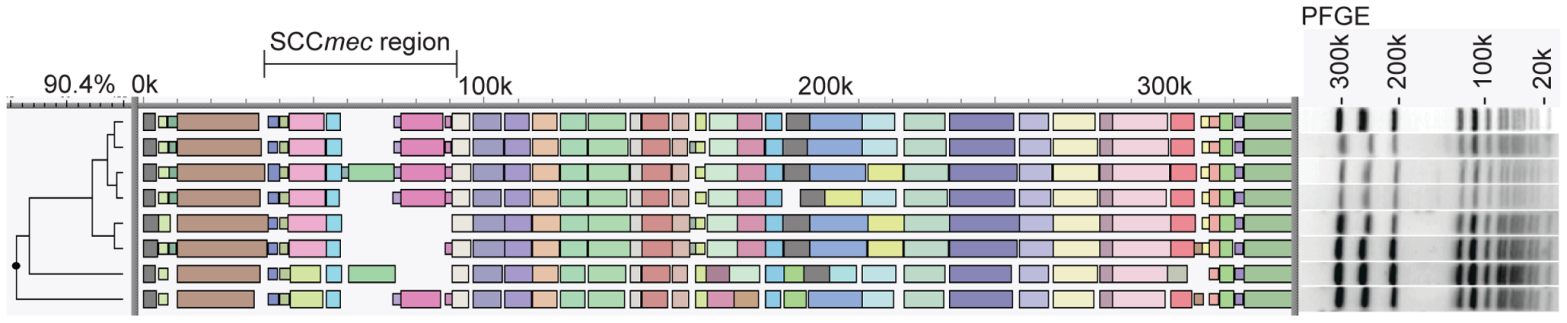
Detail of whole genome maps showing differences between HA-MRSA outbreak isolates obtained from a large medical care center in the Netherlands. Based on molecular typing (*spa*-typing, MLVA and PFGE) all isolates were indistinguishable.

The second set of isolates were presumed hospital-acquired MRSA (HA-MRSA) and originated from an outbreak in two large medical care facilities in the Netherlands that started in 2001 and persisted for a period of 20 months [20]. Since many MRSA were isolated during this outbreak, we randomly selected 8 isolates with identical genotypes (*spa*-type t445 and MT512 (MC45) and indistinguishable PFGE) from one facility for whole genome mapping. Although the WGMs of 4 of the isolates were closely related with similarities of >98.5%, there were major differences with the maps of the other isolates resulting in only 90.4% similarity between the most distinct maps (Figure 3).

### Discriminatory Power of Whole Genome Mapping for LA-MRSA

**Figure 4:**
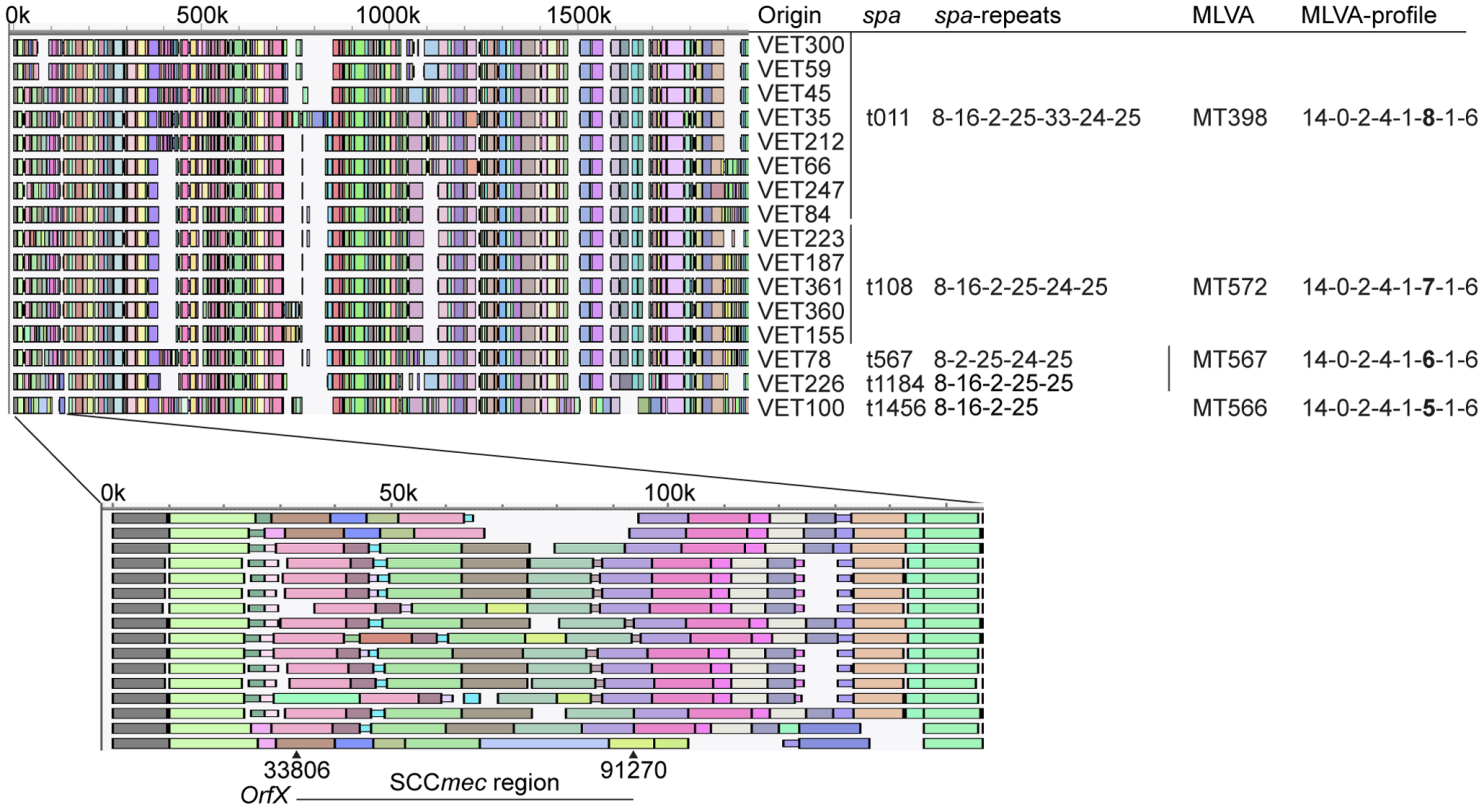
Detail of the WGMs of 16 LA-MRSA isolates originating from unrelated veterinarians showing the discriminatory power of whole genome mapping. The limited variation obtained by MLVA- and *spa*-typing is displayed on the right hand side of the WGMs. The blowup of the WGMs displays considerable variation in the SCC*mec* region.

LA-MRSA isolates obtained from 16 epidemiologically unrelated veterinarians frequently visiting livestock farms were subjected to molecular typing, including whole genome mapping. *Spa*-typing and MLVA could hardly discriminate these isolates yielding only 5 different *spa*- and 4 MLVA-types caused by variations in the number of *spa*-repeats only. In contrast, whole genome mapping was able to discriminate these isolates and the average similarity between maps ranging from 77.0% to 98.3%. However, 2 of the 16 WGMs were nearly identical, with a similarity of 99.6%. Considerable variation was seen in the SCC*mec* region of these isolates with 11 different compositions of the SCC*mec* region among the 16 isolates (Figure 4).

### Capability to Identify Transmission Events of LA-MRSA

**Figure 5:**
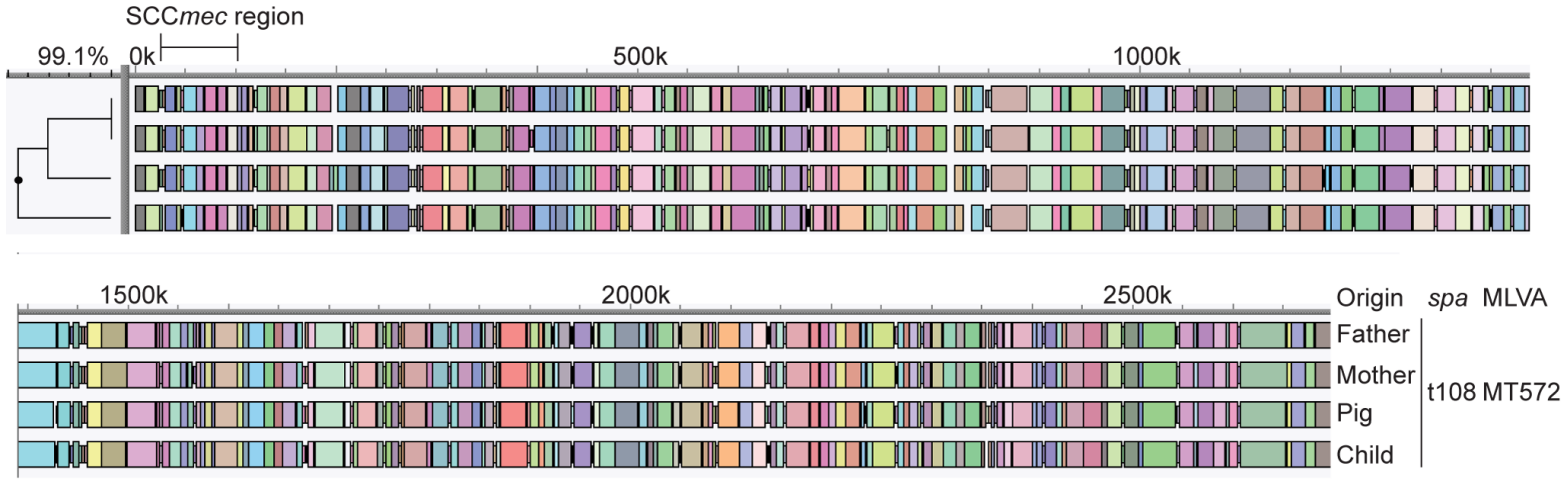
Complete WGMs of LA-MRSA isolates obtained from a confirmed transmission event. The 4 isolates represent 3 household members and 1 isolate originated from a pig on the farm. All isolates were identical in PFGE, *spa*-typing and MLVA.

We investigated the capability of whole genome mapping to identify LA-MRSA transmission events using LA-MRSA isolates obtained during a LA-MRSA outbreak that occurred in 2004 [6]. The 4 isolates used in the study presented here were obtained from a pig farmer’s family and originated from a mother suffering from LA-MRSA mastitis, from the infant that she nursed, from the farmer who is the father of the child and from one of the pigs that were sampled during the study. All isolates were *spa*-type t108, MT572 and PFGE profiles were identical. The WGMs of the 4 isolates were virtually indistinguishable yielding a similarity of 99.1%, showing that the transmission event within this family could be confirmed by WGM (Figure 5).

### WGM to Study Suspected LA-MRSA Transmission Events

**Figure 6:**
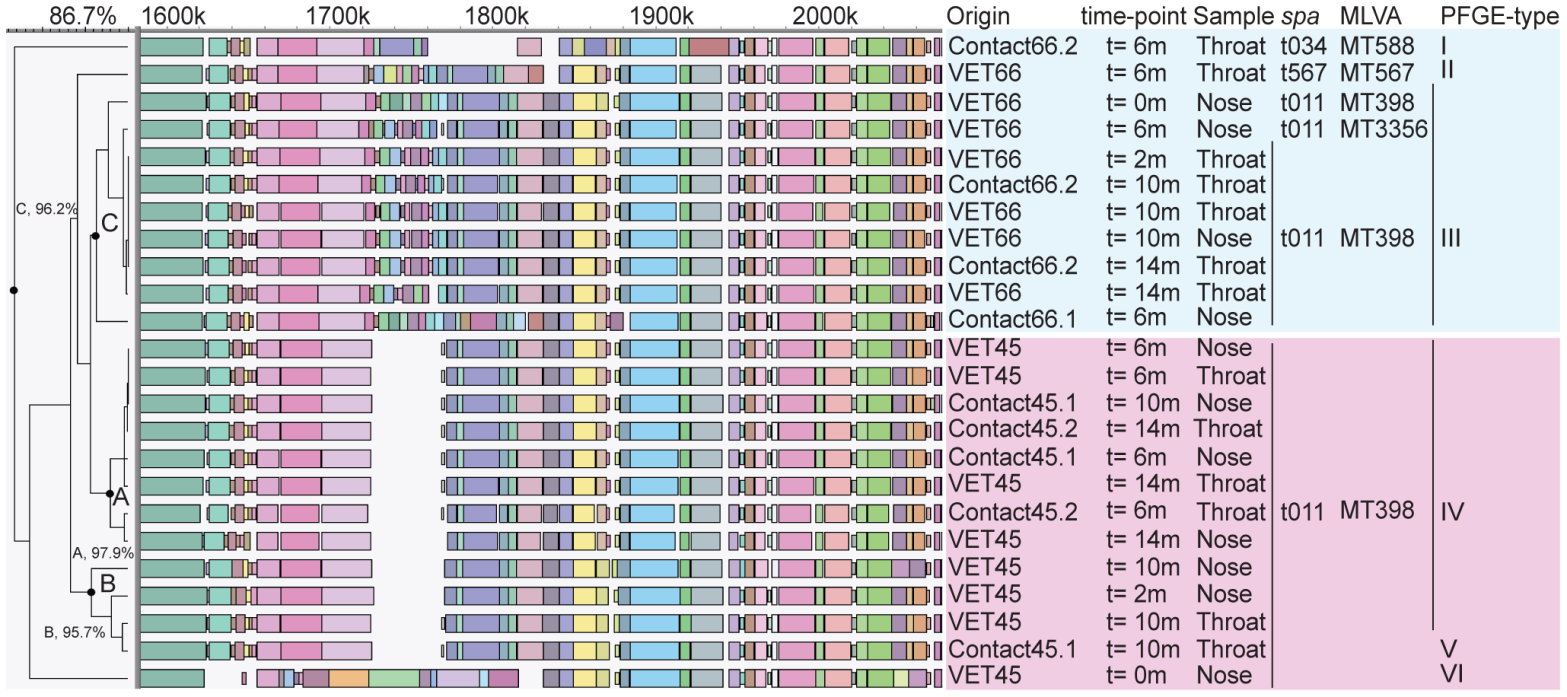
Detail of the WGMs of two veterinarians and their household members showing transmission events. A and B denote the clusters with highly similar WGMs of isolates obtained from VET45 and his household members (light red block). C denotes the cluster with highly similar WGMs of isolates obtained from VET66 and his household members (blue block). Sampling time-points, sampling sites, *spa*-type, MLVA-type and PFGE-type are indicated on the right hand side of the maps. The PFGE-type numbers are arbitrary numbers.

A set of samples originating from two families of epidemiologically unrelated veterinarians frequently visiting livestock farms that were screened for MRSA carriage on 5 time points during a fourteen-month survey period were used to assess the capability of WGM to track transmission of LA-MRSA from veterinarians to their household members. After the first screening (t = 0 m), sampling took place during home visits after 2 months (t = 2 m) and by the veterinarians themselves after 6, 10 and 14 months (t = 6 m, t = 10 m, t = 14 m). During this study period, 24 LA-MRSA isolates were cultured from the two families. Thirteen LA-MRSA isolates were obtained from veterinarian VET45 and his household members, while 11 isolates were cultured from veterinarian VET66 and his household members. *Spa*-typing and MLVA characterized 21 of 24 isolates as *spa*-type t011 and MT398 (MC398). The *spa* and MLVA profiles from the 3 other isolates, all originating from the family of VET66, differed only slightly from the 21 other isolates. PFGE yielded indistinguishable banding patterns for 11 of the 13 isolates obtained from the family of VET45 and the profiles of the other 2 isolates, differed from that of the 11 isolates in a single band and in 2 bands, respectively. Nine of the 11 isolates from VET66 and his household members had identical PFGE patterns, while the remaining 2 isolates were clearly different. The PFGE profiles of the two families represented two distinct groups, which was corroborated by WGM. Within the isolates from VET45 and his household members, two different WGM-clusters were identified. The first cluster (cluster A) was comprised of 8 isolates with a similarity of 97.9% between the most distinct WGMs and 6 of the maps were more than 99.5% similar. The second cluster (cluster B) was comprised of WGMs from 4 isolates and was distinct from the first cluster with a similarity between the first and second cluster of 93.3%. PFGE profiles of 11 of the 12 isolates belonging to these clusters were identical, showing the high discriminatory power of WGM. The remaining WGM obtained from the first isolate (t = 0 m) cultured from VET45, was quite distinct and did not belong to cluster A or cluster B. This isolate also had a distinct PFGE profile, indicating that the veterinarian carried a different strain at this time. Within the 11 LA-MRSA isolates obtained from the second family (VET66), the maps of 9 isolates were nearly identical with a similarity of 96.2% between the most distinct maps within this cluster (cluster C). These 9 isolates were also indistinguishable with PFGE. The remaining 2 isolates differed considerably in all typing analyses (Figure 6).

The sample from veterinarian VET66 taken at t = 0 m contained LA-MRSA with a WGM that was identical to those found for the LA-MRSA from the same veterinarian up to 14 months later. At sampling points from 6 to 14 months this LA-MRSA was also isolated from the household members at sampling moments 6 to 14 months. Remarkably, the veterinarian apparently carried 2 distinct LA-MRSA strains in nose and throat at the 6 month sampling point and it was the strain found in the nose that had been transmitted to its household members. In the family of VET45, WGM suggested that transmission with 2 different strains had taken place at different time points. The first strain (cluster A) was isolated from VET45 and his household members at the 6 months sampling point and thereafter. The second strain (cluster B) was isolated from VET45 at the 2 months (nose) and 10 months sampling point (nose and throat). This strain was also found in contact45.1 (HHM45.1) at 10 months (throat), but remarkably, a different strain was isolated from the same contact at the same sample moment, albeit in a different anatomic site (nose) (Figure 6).

## Discussion

LA-MRSA isolates are hard to discriminate when using current molecular typing techniques, such as *spa*-typing, MLST and MLVA. Although the PFGE using *Cfr*9I provides a much better differentiation of MC398 isolates this method is laborious and yields data that are not easily electronically exchanged. This hampers the study of possible transmission events and outbreaks caused by this MRSA clade. The whole genome mapping presented here provides a typing method with high discriminatory power that appears to be suitable to identify LA-MRSA transmission events.

The discriminatory power of WGM was illustrated by the ability to type and differentiate LA-MRSA isolates obtained from epidemiologically unrelated veterinarians frequently visiting livestock farms for which *spa*- and MLVA-typing failed to provide clear distinction. Among these unrelated isolates many different compositions of the SCC*mec* region existed indicating that variation in this locus significantly contributes to observed genomic diversity among LA-MRSA. Furthermore, WGM was able to confirm well documented CA-MRSA, HA-MRSA and LA-MRSA transmission events. In the first outbreak, involving transmission of a USA300 strain in a beauty salon, WGM identified a DNA segment of approximately 40 kb present in only 3 of the 8 isolates. Although this was not reported as such in the original paper, the additional fragment was also detected by PFGE. This additional fragment most likely represents a bacteriophage which usually has a genome size of approximately 40 kb and has the ability to jump in and out of bacterial genomes [28]. This shows that variation may occur within the same strain by gain or loss of mobile elements. Such events will lead to very localized changes whereas differences between distinct strains are the result of various genetic events and therefore in general occur scattered throughout the chromosome. This is an important criterion to decide whether two isolates may represent the same strain and thus might indicate the occurrence of a transmission event.

WGM of isolates presumably belonging to a HA-MRSA outbreak in the Netherlands revealed that several isolates did not belong to the outbreak. Although, this multi-center outbreak expanded over a long period of time and involved many different patients, the isolates we selected for this study originated from one center and were received within 2 weeks of each other. We believe that we were unable to assign all isolates as part of the outbreak because multiple strains yielding the same molecular characteristics (*spa*-typing, MLVA and PFGE) were circulating at the time of the outbreak. Indeed in 2002, 19% (*n* = 265) of all MRSA isolates sent to the RIVM had the PFGE-type that was identified as the outbreak-type. Of these isolates one-third originated from health care centers other than the two that were identified as the outbreak centers. This shows that the higher discriminatory power of WGM makes it possible to better assess whether isolates belong to an outbreak or not.

We employed WGM to assess whether the technique is suitable to identify transmission events of LA-MRSA in a community setting (i.e. transmission from veterinarians to their household members). Indeed, we obtained virtually identical WGMs of the isolates obtained from the veterinarians and their household members. However, not all isolates were identical and two different clusters were identified among the isolates of one veterinarian and his household members. These results suggest that veterinarians may pick up different LA-MRSA strains during their visits to animal farms and become colonized for a longer time period. Apparently, veterinarians may carry different LA-MRSA strains in their nose and throat at the same time and both may be transmitted to their direct contacts. We conclude that WGM now enables us to identify transmission events of LA-MRSA which would be impossible using *spa*-typing or MLVA and with much more uncertainty when using PFGE. We are currently conducting WGM of isolates obtained from a larger number of veterinarians and their household members to study LA-MRSA transmission among this group in further detail.

Based on the comparisons made of replicates of both MRSA and LA-MRSA isolates in this report and allowing for the presence of occasional mobile elements we now consider MRSA isolates with WGMs that have similarities of 98% or higher as indistinguishable. Isolates with WGMs with similarities between 95–98% may represent the same strain or should be regarded as highly related strains and those with maps that have similarities below 95% are deemed different strains. These cut-off values are supported by a recent report of Shukla et al. in which WGM of *S. aureus* was described and where a map distance of 5% was chosen as a cut-off point to define a WGM cluster [16]. This cut-off value was used for WGM of clonally related USA300 MRSA isolates using *Xba*I as restriction enzyme, but seems to be valid for LA-MRSA and other restriction enzymes as well.

We do acknowledge that based on our cut-off criteria the *in silico* maps and the maps created in our lab would not be designated as identical as would be expected. Although showing good concordance, we found inconsistencies in the size, number and order of fragments between the WGMs created in the lab and the *in silico* maps. The fragments present in the *in silico* maps were generally larger, but that should have been compensated for by the tolerance settings. The subcultures of the reference strains from which DNA was isolated to generate the whole genome sequences which were used to create the *in silico* maps and the subcultures used in our lab to create the real WGMs were not identical. Since the isolates used for comparison of *in silico* and real maps may have been subcultured for many years in various laboratories, changes in the genome may have occurred over time and this might explain for some of the observed differences. Another possible explanation could be that either the whole genome sequences of the isolates used for generating *in silico* maps contain sequence errors or that the assembly of the WGMs is inaccurate. However, the reproducibility of the WGM procedure assessed by repeated mapping of the same DNA sample on 4 consecutive days turned out to be excellent. To exclude that subculture of the reference isolates caused the observed differences we are currently assessing the complete genome sequences of 3 LA-MRSA isolates and we will compare the *in silico* maps based on these sequences with those obtained in the lab.

Molecular typing is used to characterize pathogens like MRSA in order to provide evidence that will support epidemiological studies on transmission and outbreaks. Furthermore, it is used to study changes in bacterial population structures e.g. to assess the effects of human intervention such as widespread antibiotic treatment and vaccination. WGM seems to be suitable to type LA-MRSA and identify transmission events where existing typing methods usually fail. An alternative that was not included in the analysis, but is rapidly gaining interest as typing tool, is whole genome sequencing (WGS). WGS has many advantages over WGM e.g. it will be very difficult to infer phylogenetic relationships and assess population structures using WGM, whereas WGS is very well suited for these purposes. Furthermore, sequencing can potentially reveal all details on gene composition, the presence of virulence factors, etc. Although we believe that WGS is the ultimate typing method, there may be a number of drawbacks for outbreak investigations leaving a niche for methods like WGM. WGS has been suggested as the best tool for typing during outbreak investigations and a number of papers have been published supporting this claim [29]–[32]. However, these studies are all retrospective investigations and they do not yet show the utility of real time WGS. Due to the relatively simple data analysis, WGM can provide a comparison with other maps within 2 days after the bacterial culture has been received, making it suitable to investigate real time transmission events and outbreaks involving pathogens such as LA-MRSA. Furthermore, the generated WGM data can be stored as a table containing the ordered restriction fragments e.g. in. xml-format and therefore are easy accessible, allowing users to quickly exchange data and compare isolates.
